# Low sodium availability in hydroponically manipulated host plants promotes cannibalism in a lepidopteran herbivore

**DOI:** 10.1038/s41598-023-48000-z

**Published:** 2023-11-27

**Authors:** Luis Y. Santiago-Rosario, Ana L. Salgado, Diego Paredes-Burneo, Kyle E. Harms

**Affiliations:** 1 Department of Biological Sciences, Louisiana State University, Baton Rouge, LA USA; 2 Department of Ecology, Evolution and Behavior, University of Minnesota, Saint Paul, MN USA; 3 Departamento de Dicotiledóneas, Museo de Historia Natural UNMSM, Av. Arenales 1256, Jesús María, Lima, Peru

**Keywords:** Behavioural ecology, Entomology

## Abstract

As an abundant element in the Earth’s crust, sodium plays an unusual role in food webs. Its availability in terrestrial environments is highly variable, but it is nonessential for most plants, yet essential for animals and most decomposers. Accordingly, sodium requirements are important drivers of various animal behavioural patterns and performance levels. To specifically test whether sodium limitation increases cannibalism in a gregarious lepidopteran herbivore, we hydroponically manipulated *Helianthus annuus* host plants' tissue-sodium concentrations. Gregarious larvae of the bordered patch butterfly, *Chlosyne lacinia*, cannibalized siblings when plant-tissue sodium concentrations were low in two separate experiments. Although cannibalism was almost non-existent when sodium concentrations were high, individual mortality rates were also high. Sodium concentration in host plants can have pronounced effects on herbivore behaviour, individual-level performance, and population demographics, all of which are important for understanding the ecology and evolution of plant-animal interactions across a heterogeneous phytochemical landscape.

## Introduction

Sodium plays a relatively unusual role within ecosystems as a nonessential element for most plants, yet as a critically essential element for animal consumers and most decomposers (e.g., bacteria, fungi)^1–4^. In most cases, plants are unable to entirely prevent the uptake of sodium from the substrate, which has important consequences for plant fitness, through its physiological effects, and by impacting community assembly and structure across space and time^5,6^. As an essential element for animals, sodium plays an important role in osmoregulatory processes, promotes muscle and neural tissue development, and controls blood movement, among other functions^7–9^. Posing challenges for animal foragers, the availability of sodium across terrestrial environments (e.g., soils and plants) is highly heterogeneous^10–13^.

Overall environmental variation of available sodium and stoichiometric mismatch (i.e., differences between what plants have and what animals need) influence animal behaviour and performance, especially among herbivorous or non-carnivorous animals, since animal prey provides sufficient sodium^11,14^. For instance, in sodium-deficient habitats, insufficient dietary sodium promotes geophagy (e.g., salt lick visitation)^14–16^ and uptake of mineral-rich water (e.g., puddling)^17–20^, causes selective consumption of sodium-rich plant tissues^21–24^, induces migratory behaviour and changes in animal population density^25,26^, and can promote a shift in omnivorous diets toward higher trophic levels^27–29^. The craving for sodium appears to be shared by many terrestrial and aquatic animals, representing a conserved condition, especially for strict primary consumers whose dietary needs rely solely on primary producers (strict herbivores and many detritivores)^8,30–32^.

Cannibalism, the consumption of conspecifics, occurs in several non-carnivorous insects^33^. For instance, sodium-deficient artificial diets increased the incidence of cannibalism in the grasshopper *Melanopus differentialis* (Orthoptera: Acrididae)^34^ and the cotton bollworm *Helicoverpa armigera* (Lepidoptera: Noctuidae), mainly when larger individuals encountered smaller conspecifics^35^ . Cannibalism is not taxonomically restricted^36–38^ and can occur in either a density-dependent or density-independent manner^33,39^. Cannibalism is known to occur in certain cases in which there is a protein or salt deficiency^25^, for acquisition of defensive chemicals^38^, during parental care^40–42^, and during courtship and reproduction^43^. Theoretical models predict that whether or not cannibalism occurs among taxa results from the relative balance between costs (e.g., increasing risk of injury and loss of inclusive fitness when harming siblings) and benefits (e.g., reducing the prevalence of parasites, intraspecific competition, or nutritional scarcity)^33,44,45^.

**Figure 1:**
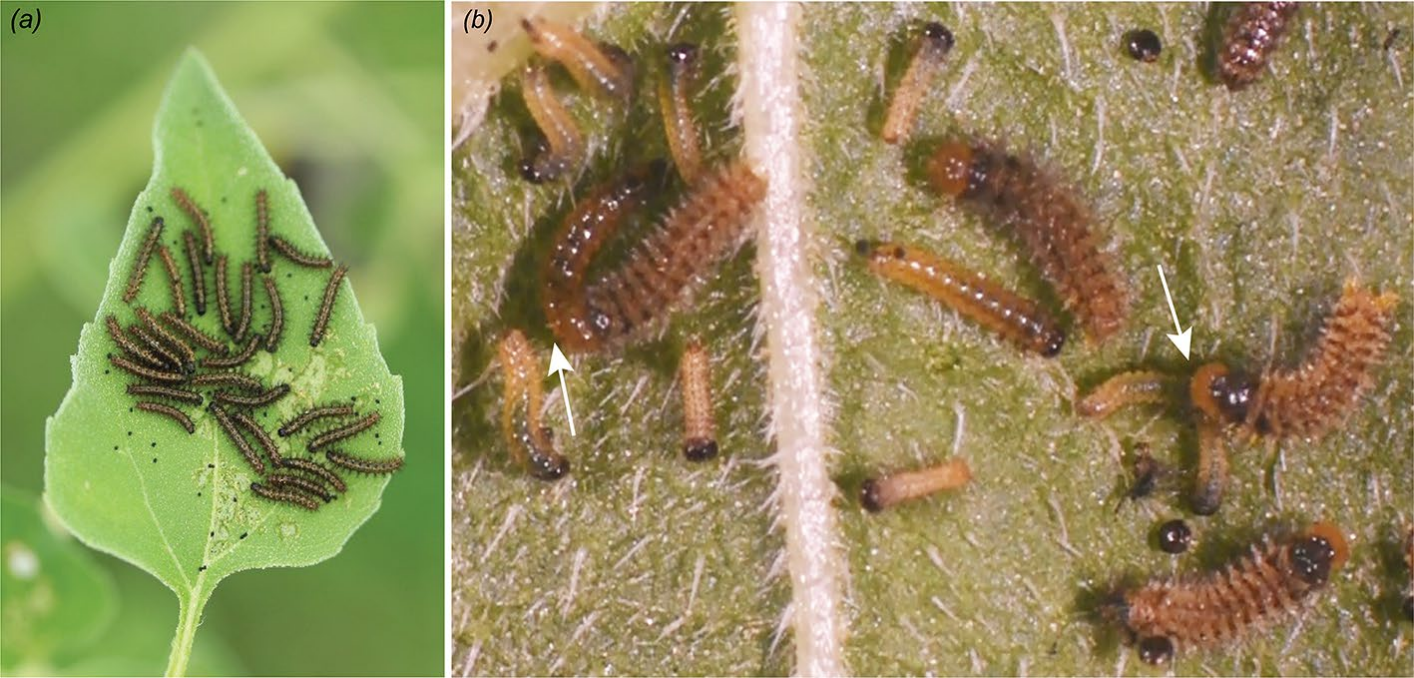
Larvae of *Chlosyne lacinia* in natural and laboratory settings. (**a**) Sibling larvae of *C. lacinia* feeding gregariously on a leaf of common wild sunflower (*H. annuus*) in southern Texas. (**b**) Larvae cannibalizing conspecifics in preliminary observations separate from our experiments using a No addition treatment. White arrows indicate two cases of cannibalism. Photos taken by Luis Y. Santiago-Rosario.

Previous studies that have evaluated the influence of dietary sodium on cannibalism and performance in insect herbivores have mostly applied sodium treatments in artificial diets or in solutions sprayed onto the foliage^34,35,46^. To our knowledge, no study of insect herbivore behaviour or performance has manipulated plant-tissue-incorporated sodium concentrations. Our hydroponic method aimed to present variation of dietary sodium in host tissue as the herbivore has experienced it in nature, i.e., they do not feed on artificial diets nor generally encounter sodium sprayed onto leaves in the field, except in coastal habitats through aerosol deposition^10^. We manipulated tissue sodium of common sunflowers (*Helianthus annuus,* Asteraceae) to test whether plant sodium concentration would influence an herbivore's cannibalistic behaviour and survival. We hypothesized that: (1) cannibalism would be higher at lower plant sodium concentrations, owing to sodium deficiency at low dietary levels; and (2) larval survival would suffer under plant sodium extremes, owing to physiological stress at the very lowest and highest levels, following a subsidy-stress response^12,47^. To test these predictions, we used the bordered patch butterfly, *Chlosyne lacinia* (Lepidoptera: Nymphalidae), a highly variable species with an extensive geographic range from Oklahoma, USA, to northern Argentina^48–52^ (Fig. 1a). The larvae are oligophagous herbivores on species of the tribe Heliantheae (Asteraceae), which can be found from coastal areas with high sodium availability to inland sodium-deficient habitats^53,54^. Females of *C. lacinia* oviposit large egg clusters (25–450 eggs) on the undersides of host plant leaves, and larvae feed gregariously, producing skeletonized leaf damage (Fig. 1a)^55–57^. Larvae of *C. lacinia* have been observed to cannibalize eggs^58^, other larvae^56^ (i.e., pg. 138, "Predation among individual larvae of the same brood"), and pupae (J. Phelps, pers. comm.). We also observed some larger larvae feeding on smaller (presumably more recently emerged) siblings in previous experiments (Fig. 1b) and as captured in a video recording (Supplementary Information S1).

## Results

### Sunflower tissue sodium increased as hydroponic-solution sodium increased

**Figure 2:**
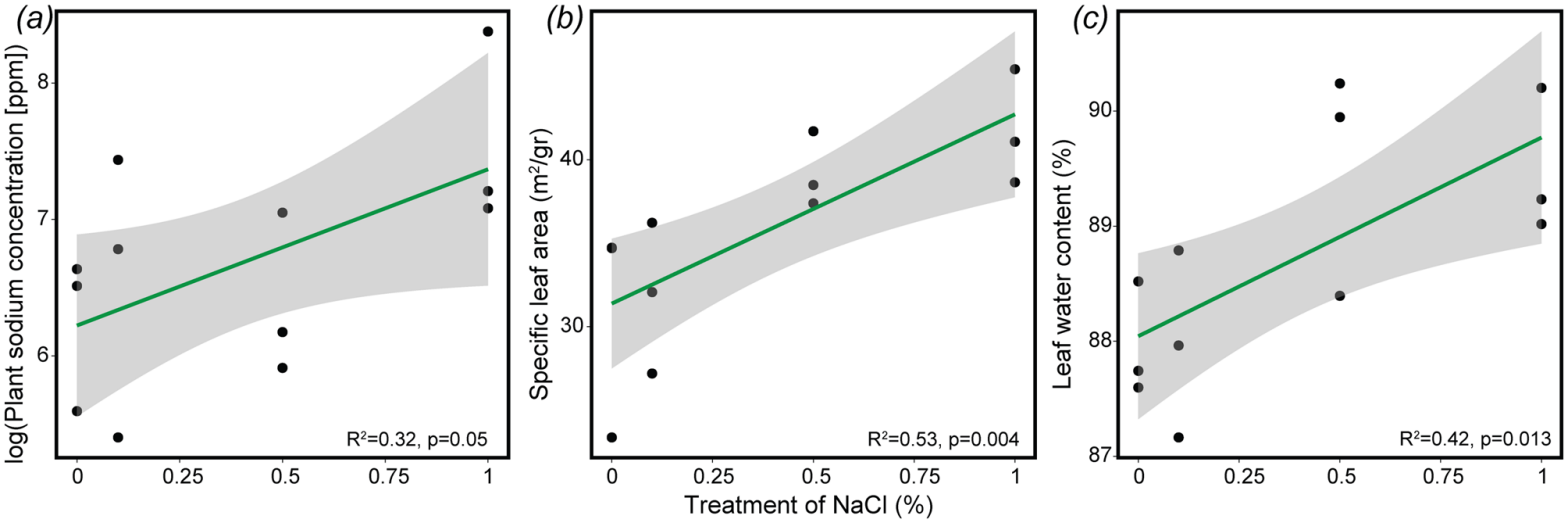
*Helianthus annuus* responses to increasing hydroponic sodium treatments. Linear regressions of (**a**) plant-sodium concentration (ppm), (**b**) specific leaf area (m^2^/g), and (**c**) leaf water content (%) as a function of treatment of NaCl (%) in the plants' hydroponic solutions.

Sodium concentrations in sunflowers increased across hydroponic-NaCl treatments (No addition = 568 ± 215 ppm; Low = 668 ± 346 ppm; Medium = 934 ± 603 ppm; and High = 2296 ± 1451 ppm) (F_(1,10)_ = 4.62, R^2^ = 0.32, *p* = 0.05, Fig. 2a), supporting the no-escape-from-sodium hypothesis, which posits that most plants’ tissues become saltier in saltier substrates^5^. Specific leaf area (m^2^/g) (F_(1,10)_ = 13.33, R^2^ = 0.53, *p* = 0.004, Fig. 2b) and leaf water content (%) (F_(1,10)_ = 8.973, R^2^ = 0.42, *p* = 0.013, Fig. 2c) also increased as sodium in the hydroponic solution increased. The values for plant-sodium concentrations across treatments fall within the range of concentrations observed for sunflower foliar tissues in the wild and across the geographic range of *C. lacinia* (minimum = 3 ppm, maximum = 29,791 ppm)^10^. We found no significant differences in plant-tissue concentrations for Ca, C, Cu, Fe, Mg, Mn, N, P, K, S, and Zn, none of which varied among the hydroponic solutions used to produce the host plants (Supplementary Table S2).

### Larval behaviour and mortality outcomes were contingent upon plant-sodium concentrations

**Figure 3:**
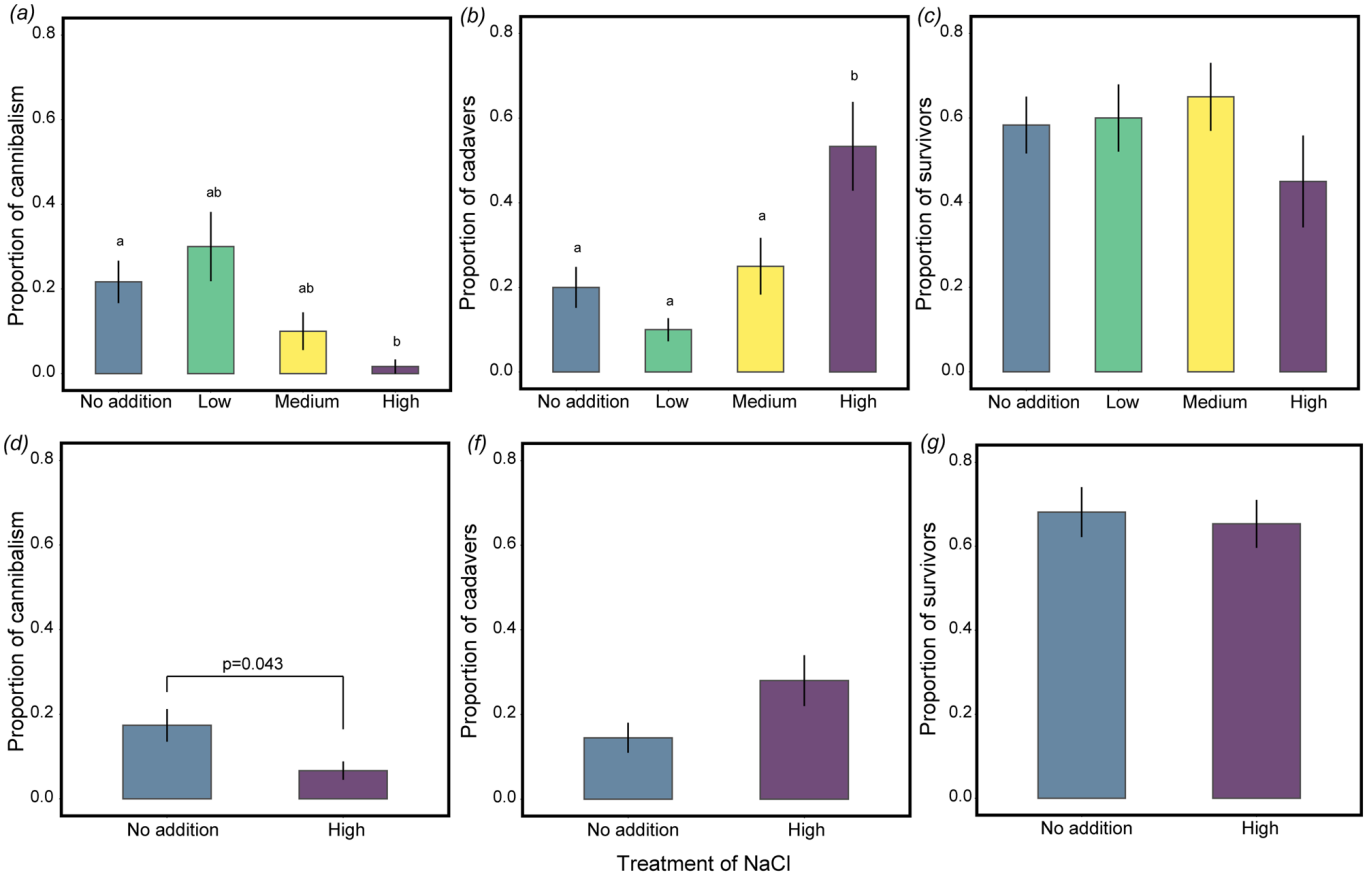
Larval responses to increasing plant hydroponic-sodium treatments. (**a**) Proportion of cannibalized, (**b**) cadavers, and (**c**) survivors, for the first experiment. Different letters indicate significant differences based on posthoc Tukey tests. (**d**) Proportion of cannibalized, (**e**) cadavers and (**f**) survivors, for the second experiment. Colours depict sodium concentration treatments: No addition (0%, blue), Low (0.1%, green), Medium (0.5%, yellow), and High (1.0%, purple). The vertical lines indicate standard errors.

To evaluate the influence of plant sodium concentration variation on larval behaviour and mortality, we conducted two experiments designed to characterize larval responses to dietary sodium concentrations. On average, *C. lacinia* larvae exhibited contrasting behavioural and mortality responses among the host-plant sodium concentrations. For the first experiment, the proportion of cannibalism peaked at the lowest concentrations (GLM: *D* = 12.02, *df* = 3, *p* < 0.001, Fig. 3a). Cannibalism was only observed once in the High treatment (1.7%). In contrast, in the No addition and Low treatments, cannibalism occurred at higher frequencies. Post-hoc analysis also confirmed the higher cannibalism frequencies in the Low as compared to the High treatment. Within the cannibalized cohort, 51.7% were distributed across the No Addition (21.7%) and Low (30%) treatments. Larval mortality resulted from two different mechanisms: cannibalism (counted as not present) and death from other causes (counted as cadavers). Most cadavers were found in the High treatment (53.3%), suggesting a substantial mortality response to dietary sodium-induced stress (GLM: *D* = 12.02, *df* = 3, *p* < 0.001, Fig. 3b). Post-hoc analysis revealed significant increase in the proportions of cadavers in the High treatment as compared with the other treatments. Larval survival per treatment was 58.3% No addition, 60% Low, 65% Medium, and 45% High treatment (Fig. 3c).

The results of the No addition versus the High sodium treatment for the second experiment were similar to those from the first experiment. The proportion of cannibalized larvae was highest at the lowest sodium concentration (GLMM: *X*^*2*^ = 4.06, *df* = 1, *p* = 0.043, Fig. 3d). Cannibalism killed 17.4% of larvae for the No addition and 6.7% for the High treatment. Larval mortality was also highest in the High treatment (28%) compared to the No addition treatment (14.5%), however, the GLMM was not significant for this comparison (*X*^*2*^ = 3.16, *df* = 1, *p* = 0.075) (Fig. 3e). Comparing the number of larvae that survived until pupation, we found that in the No addition 63.1% survived and in the High treatment 65.3% (Fig. 3f).

## Discussion

Most terrestrial animals have evolved in environments in which sodium is scarce. Thus, an intricate network of mechanisms has evolved for terrestrial and freshwater aquatic animals to regulate sodium within their bodies and tissues^59,60^. When found in excess, animals must overcome sodium toxicity, generally through excretion, which is energetically costly ^7,61,62^. We found that the highest sodium levels resulted in the highest mortality, most likely due to osmotic, physiological stress^62^ and/or as a consequence of other plant responses to variation in salinity (e.g., variation in leaf toughness, secondary chemical profiles, etc.)^63,64^. Similar responses have been observed in other lepidopteran species. For instance, monarch butterfly (*Danaus plexippus,* Lepidoptera: Nymphalidae) larvae experienced high mortality when exposed to milkweed (*Asclepias*, Apocynaceae) collected near roadsides in Minnesota, where salts are used for deicing, with plants having 16 times higher sodium concentration than plants collected in natural habitats away from roads^46^.

Supporting the idea of stoichiometric mismatch, we found that limited dietary sodium in plant tissues was associated with cannibalism in a species that feeds gregariously during the larval stages. The possibility of additional contributing factors cannot be definitively excluded, as certain traits in hydroponic plants exhibited correlated responses to sodium, *i.e.*, specific leaf area and leaf water content. Although rare across the tribe Melitaeini (Lepidoptera: Nymphalidae), cannibalism has been reported in *C. lacinia* by 1^st^ instar larvae on eggs and among larvae, especially in crowded conditions^56,58^. In our case, larval groups for each replicate consisted of siblings in each of two separate experiments. Since cannibalism occurred among siblings, their craving for sodium appears to have been particularly powerful, *i.e.,* to overcome the inclusive fitness losses, when the plant host proved sodium deficient. Density also plays an important role in triggering cannibalism in some species^65,66^. If cannibalism occurs in a density-dependent manner in *C. lacinia*, it may be that the elevated mortality through causes other than cannibalism in the Medium and High treatments reduced numbers of individuals sufficiently to concomitantly reduce observed levels of cannibalism in those treatments. However, this explanation is unlikely because cannibalism across all treatments generally occurred at the earliest life-cycle stages when larval densities among treatments were on average the same.

In conclusion, low plant-sodium concentration was associated with increased cannibalism in larvae of *C. lacinia*, an otherwise non-carnivorous, gregariously feeding herbivore. Mortality from other causes was elevated at the highest levels of plant-tissue sodium, presumably owing to sodium toxicity and/or perhaps resulting from other unintended but correlated plant responses to high hydroponic-solution sodium. Therefore, our study suggests that plant-sodium concentrations can have variable and important influences on animal behaviour and performance. Sources of sodium for animal consumers are highly variable across the terrestrial phytochemical landscape^10,67,68^, which can be consequential for micro- and macroevolutionary processes, local adaptation, community assembly, food web structure, and eco-evolutionary dynamics.

## Methods

### Hydroponics and plant-sodium concentrations

We grew domesticated common sunflower, Sunspot cultivar (Urban Farmer https://www.ufseeds.com), across sodium-concentration treatments using a hydroponic floating raft method^69^. Plants were grown using a full spectrum light regime of 16L/8D, at a temperature of 23 ± 3 ºC, and relative humidity of 60 ± 10% for a month. Seeds were sown in groups of six in 9-oz Styrofoam cups filled with 2:1 parts sand and vermiculite (Uline https://www.uline.com/) and were kept in 49 L trays with 16 L of hydroponic solution. Hoagland’s modified basal salt mixture was used as the hydroponic solution (1 M Ca(NO_3_)_2_.4H_2_O, 1 M KNO_3_, 1 M NH_4_H_2_PO_4_, 1 M MgSO_4_.7H_2_O, 9.2 mM MnCl_2_.4H_2_O, 0.77 mM ZnSO_4_.7H_2_O, 0.32 mM CuSO_4_.5H_2_O, 0.11 mM MoO_3_, 90 mM FeSO_4_.7H_2_O, and 0.5 M H_3_BO_3_) supplied weekly at half strength^70,71^. Once a week, trays were purged of the hydroponic solution and replenished with new solutions and treatments. Water levels were kept constant with distilled water. For the first experiment, our sodium treatments were: No addition (0% NaCl); Low (0.1% NaCl); Medium (0.5% NaCl); and High (1.0% NaCl). We used three trays for each treatment, and within each tray there were 36 individual pots. Leaves of five plants per tray were pooled and sent for plant-chemistry analysis for Ca, C, Cu, Fe, Mg, Mn, N, P, K, Na, S, and Zn using Inductively Coupled Plasma—Atomic Emission Spectrometry (ICP-AES)^72^. Plant elemental concentrations (ppm) across hydroponic treatments were analysed using linear regression. A second experiment followed the same plant-growth protocol, but we used only the No addition and High treatments of sodium in 25 trays per treatment. Two trays in the No addition treatment were excluded because plants died. The seed material and plants used for this study complied with relevant local, institutional, national, and international guidelines, permissions, or legislation. No permits were required to perform this study.

### Butterfly colony

For our first experiment, we collected larvae and adult individuals (i.e., F0) from Dilley, TX (28.66, − 99.22), Sullivan City, TX (26.27, − 98.56), and Mission, TX, USA (26.17, − 98.33) in May 2021. Individuals were reared on sunflower plants (Mammoth Grey cultivar, Papaw’s Garden Supply https://papawsgarden.com/). Larvae (i.e., F1) were reared in mesh cages on sunflower plants in the following conditions: light regime of 16L/8D, at a temperature of 23 ± 3 ºC, and relative humidity of 60 ± 10%^49^. After the first generation, adult butterflies were mixed across rearing cages to increase genetic diversity and reduce inbreeding depression for subsequent generations. We randomly selected 1st-instar clusters (each cluster constituted a group of half-siblings) of the F2 generation for the feeding trials. In the second experiment, we collected larvae from the same locations in September 2022. Larvae were fed ad libitum with sunflower leaves and reared until adulthood. Once adults, these F0 individuals were mixed for mating, and oviposition occurred on sunflower plants. We chose four larval clusters of the F1 to set up the second feeding trials to reduce the chances of larvae altering their behaviour due to prolonged artificial lab rearing.

### Larval exposure to dietary sodium and data analysis

In both experiments, groups of six sibling larvae were exposed to dietary sodium treatments. For each group, individuals were selected at random from a single 1st-instar cluster from the colony. Each group was added to a 32-oz Styrofoam cup containing ad libitum sunflower plants grown on the randomly assigned salt-concentration treatment. Plants were randomly chosen from three trays designated for each treatment to replenish after larvae completed feeding entirely on foliar tissues. Larvae were kept on live plants until death or pupation. For the first experiment, each sodium treatment had ten group replicates (total of 40 groups). The study lasted 21 days. We observed each replicate every day, and counted individuals every three days to determine the number of larvae alive at each stage, the number of individuals cannibalized (i.e., the absence of the larval body was inferred to result from cannibalism, because thorough and frequent checks of the containers rendered it highly unlikely that a dead body could have sufficiently rapidly decayed to be missed or predated by an unobserved predator) and the number of larval cadavers (which died of causes other than cannibalism; determined by the presence of a body that was not moving). We followed the same protocol for the second experiment, with 23 larval groups in the No addition and 25 in the High treatment (a total of 288 individuals). In addition, for the second experiment, we also kept track of broods, to include this information in the statistical model. To reduce the influence of unintended observer biases, three researchers counted the number of larvae per treatment for both experiments.

To test the influence of plant-sodium concentrations on the proportion of individuals lost to cannibalism and death by other causes, we performed a generalized linear model (GLM), for the first experiment. Sample units were the groups of six larvae, i.e., cups. We included plant sodium concentration as a fixed factor (categorical) because a preliminary analysis using the mean sodium concentrations of the plants gave similar results as using categorical classifications. For the second experiment, we used a generalized linear mixed model (GLMM). We included plant sodium concentration as a fixed factor (categorical), and clutch ID as a random factor to account for potential lineage differences. The models were analysed using a beta-binomial distribution and the *glm* and *glmmTMB* functions of the packages *stats*^73^ and *glmmTMB*^74^*,* respectively. Each model's performance was inspected by plotting residuals against fitted values and confirming that the models were not overdispersed. To estimate the effects of plant-sodium concentration on larval fate of the first experiment, we used a Tukey pairwise contrast^75^. All analyses were performed in R 4.2.0^73^.

### Supplementary Information

Supplementary Table S2.

Supplementary Video 1.

Supplementary Table S2 legend.

Supplementary Video legend.

## Data Availability

Data used in this study is available in the Dryad Digital Repository: https://doi.org/10.5061/dryad.qjq2bvqjk.
