# Supplementary material for: Low sodium availability in hydroponically manipulated host plants promotes cannibalism in a lepidopteran herbivore
Source: Sci Rep. 2023 Nov 27;13:20822. doi: 10.1038/s41598-023-48000-z (PMC10682487; doi:10.1038/s41598-023-48000-z)
Supplement: Supplementary file 1 — Supplementary Table S2. [file 41598_2023_48000_MOESM1_ESM.docx]

Supplementary Table S2:

| **Plant element measured** | **Treatment of NaCl** | **Mean concentration (ppm)** | **Standard error** | **Linear regression** |
| --- | --- | --- | --- | --- |
| Calcium (Ca) | No addition | 16,862 | 3,889 | F_(1,10)_=0.16, R^2^=0.02, p=0.70 |
|  | Low | 15,547 | 1,546 |  |
|  | Medium | 18,778 | 2,506 |  |
|  | High | 15,457 | 5,362 |  |
| Carbon (C) | No addition | 416,651 | 6,297 | F_(1,10)_=2.45, R^2^=0.19, p=0.15 |
|  | Low | 399,495 | 9,583 |  |
|  | Medium | 406,522 | 4,538 |  |
|  | High | 397,712 | 6,808 |  |
| Copper (Cu) | No addition | 7.4 | 1.43 | F_(1,10)_=0.19, R^2^=0.02, p=0.68 |
|  | Low | 7.7 | 0.63 |  |
|  | Medium | 7.1 | 1.22 |  |
|  | High | 7.3 | 2.19 |  |
| Iron (Fe) | No addition | 142 | 40.3 | F_(1,10)_=0.21, R^2^=0.02, p=0.65 |
|  | Low | 100 | 15.9 |  |
|  | Medium | 122 | 23.1 |  |
|  | High | 143 | 53.6 |  |
| Magnesium (Mg) | No addition | 6,742 | 883 | F_(1,10)_=0.09, R^2^=0.01, p=0.76 |
|  | Low | 6,705 | 130 |  |
|  | Medium | 7,242 | 854 |  |
|  | High | 6,497 | 1,180 |  |
| Manganese (Mn) | No addition | 57 | 6.5 | F_(1,10)_=4.09, R^2^=0.29, p=0.07 |
|  | Low | 53 | 4.3 |  |
|  | Medium | 67 | 8.1 |  |
|  | High | 77 | 21.9 |  |
| Nitrogen (N) | No addition | 44,125 | 5,457 | F_(1,10)_=2.05, R^2^=0.17, p=0.18 |
|  | Low | 44,696 | 1,941 |  |
|  | Medium | 44,821 | 1,482 |  |
|  | High | 49,018 | 4,968 |  |
| Phosphorous (P) | No addition | 5,626 | 1,258 | F_(1,10)_=0.55, R^2^=0.05, p=0.48 |
|  | Low | 5,955 | 456 |  |
|  | Medium | 5,129 | 760 |  |
|  | High | 6,659 | 1,328 |  |
| Potassium (K) | No addition | 53,864 | 10,643 | F_(1,10)_=0.22, R^2^=0.02, p=0.65 |
|  | Low | 56,535 | 1,649 |  |
|  | Medium | 48,363 | 4,670 |  |
|  | High | 58,616 | 2,776 |  |
| Sodium (Na) | No addition | 568 | 214 | F_(1,10)_=4.62, R^2^=0.32, **p=0.05** |
|  | Low | 668 | 346 |  |
|  | Medium | 933 | 603 |  |
|  | High | 2,295 | 1,451 |  |
| Sulfur (S) | No addition | 5,415 | 259 | F_(1,10)_=0.06, R^2^=0.01, p=0.81 |
|  | Low | 5,287 | 231 |  |
|  | Medium | 5,100 | 470 |  |
|  | High | 5,339 | 340 |  |
| Zinc (Zn) | No addition | 18.9 | 5.8 | F_(1,10)_=1.71, R^2^=0.15, p=0.22 |
|  | Low | 16.6 | 4.1 |  |
|  | Medium | 17.5 | 4.1 |  |
|  | High | 23.1 | 3.4 |  |
