# Supplementary material for: Low sodium availability in hydroponically manipulated host plants promotes cannibalism in a lepidopteran herbivore
Source: Sci Rep. 2023 Nov 27;13:20822. doi: 10.1038/s41598-023-48000-z (PMC10682487; doi:10.1038/s41598-023-48000-z)
Supplement: Supplementary file 3 — Supplementary Table S2 legend. [file 41598_2023_48000_MOESM3_ESM.docx]

**Supplementary:**

Supplementary Table S2: Elemental mean concentrations (and standard errors) across plants in the first experiment. A linear regression was performed for each element to test the differences in elemental concentrations across treatments.
