# Supplementary material for: Low sodium availability in hydroponically manipulated host plants promotes cannibalism in a lepidopteran herbivore
Source: Sci Rep. 2023 Nov 27;13:20822. doi: 10.1038/s41598-023-48000-z (PMC10682487; doi:10.1038/s41598-023-48000-z)
Supplement: Supplementary file 4 — Supplementary Video legend. [file 41598_2023_48000_MOESM4_ESM.docx]

**Supplementary:**

Supplementary Information S1: Video depicting cannibalism instance by two 2^nd^-instar larvae of *Chlosyne lacinia* on smaller conspecifics.
